# Post-synthetic modified luminescent metal–organic framework for the detection of berberine hydrochloride in a traditional Chinese herb†

**DOI:** 10.1039/d3ra07054a

**Published:** 2024-01-02

**Authors:** Wei Liu, Shuang Wu, Tian-Xia Sun, Jing Bai, Ying Yang, Wen-Hui Lian, Yu Zhao

**Affiliations:** a College of Pharmacy, Changchun University of Chinese Medicine Changchun 130017 P. R.China; b Jilin Ginseng Academy, Changchun University of Chinese Medicine Changchun 130017 P.R.China lianwenhui1014@126.com cnzhaoyu1972@126.com; c Jilin Ji Test Technology Co. LTD Changchun 130017 P. R.China

## Abstract

In this work, a novel fluorescence sensor UiO-66-PSM based on post-synthetic modified metal–organic frameworks was prepared for the detection of berberine hydrochloride (BBH) in the traditional Chinese herb *Coptis*. UiO-66-PSM was synthesized by a simple Schiff base reaction with UiO-66-NH_2_ and phthalaldehyde (PAD). The luminescence quenching can be attributed to the photo-induced electron transfer process from the ligand of UiO-66-PSM to BBH. The UiO-66-PSM sensor exhibited fast response time, low detection limit, and high selectivity to BBH. Moreover, the UiO-66-PSM sensor was successfully applied to the quantitative detection of BBH in the traditional Chinese herb *Coptis*, and the detection results obtained from the as-fabricated fluorescence sensing assay were consistent with those of high-performance liquid chromatography (HPLC), indicating that this work has potential applicability for the detection of BBH in traditional Chinese herbs.

## Abstract 2

The fluorescence sensor UiO-66-PSM was prepared by post-synthetic modification. Fluorescence properties of UiO-66-PSM confirmed that the prepared sensor can be applied to the detection of berberine hydrochloride the in traditional Chinese herb *Coptis*.

## Graphical abstract

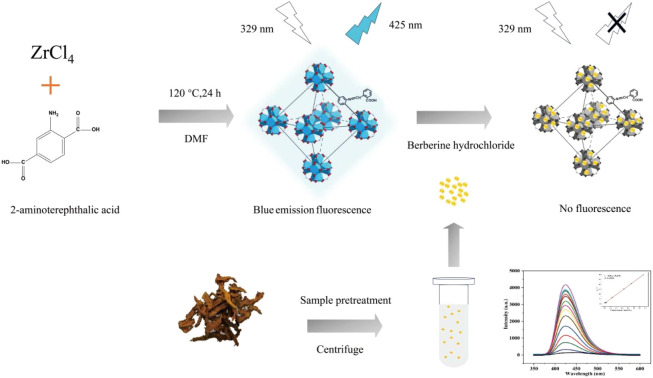

## 1. Introduction

Berberine hydrochloride (BBH) is an isoquinoline alkaloid extracted from traditional Chinese herbs *Coptis*, *Cortex phellodendri*, *etc.* A large number of studies have shown that BBH has a variety of pharmacological effects,^1^ including anti-pathogenic microorganisms, anti-inflammatory, antitumour, cardioprotective, hypoglycaemic, lipid metabolism regulation, and immunosuppression. Various methods have been reported for the detection of BBH, including chromatography,^2^ colorimetry,^3^ electrochemistry,^4^ and fluorescence.^5^ Compared with other methods, fluorescence is a favourable method for the analysis of BBH due to its simple operation, high sensitivity, and better selectivity. To date, several fluorescent sensors have been developed for the detection of BBH, including carbon dots,^6^ quantum dots,^7^ metal nanoclusters^8,9^ and metal–organic frameworks (MOFs).^10^ MOFs have been widely used in sample pretreatment,^11–13^ catalysis,^14^ drug delivery,^15^ fluorescence bioimaging,^16^ and electrochemical and fluorescence sensing^17–19^ based on their controlled pore size, large specific surface area and tunable molecular structure. However, as far as we know, only a handful of MOFs-based fluorescent sensors have been applied to the detection of ingredients in traditional Chinese herbs.^20–23^ A literature survey indicates that MOFs-based fluorescence sensors used for the detection of BBH are sparse except for the following report. Xiong *et al.*^10^ prepared a microscale highly fluorescent Eu metal–organic framework, which was used as a dual-mode visual sensor for the sensitive detection of BBH and tetracycline. Therefore, it is very significant to develop MOFs-based materials for highly sensitive and selective detection of BBH.

Luminescent metal–organic frameworks (LMOFs), as a kind of MOFs materials, have attracted increasing attention from scientific researchers, especially in the field of fluorescence sensing.^24–26^ LMOFs sensors have made some progress in the detection of anions,^27^ cations,^28^ volatiles and gas molecules.^29^ However, it is still a great challenge to prepare an ideal MOFs structure with the desired performance and functionality. Direct doping and post-synthetic modification (PSM) have become effective strategies to overcome this problem;^30,31^ in particular, PSM has been widely applied in the sensing field because it only occurs on the frame or within the aperture, and does not damage the crystal structure. For instance, in our group's previous work, Yu *et al.*^32^ synthesized a highly luminescent MOF (TMU-PC) by covalent PSM of TMU-17-NH_2_ with 2-pyridinecarboxaldehyde for rapid detection of inorganic pyrophosphate in human urine and synovial fluid samples.

As a typical representative of the LMOFs family, UiO-66 series functional materials have been applied in fluorescence sensing based on the advantages of large specific surface area, unsaturated metal sites, functionalized framework structure, good stability, *etc.*^33,34^ The most common is UiO-66-NH_2_ with a weak blue fluorescence, which is prepared by organic ligand containing chromophore amino group.^35^ The amino group in UiO-66-NH_2_ is not only an active but also a basic group, which can undergo condensation reaction with the active carbonyl group *via* covalent PSM, *i.e.* Schiff base reaction.^36^

**Scheme 1:**
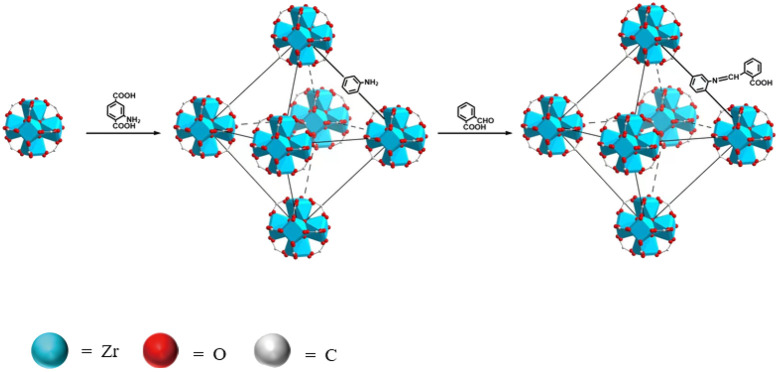
Synthesis process of UiO-66-PSM.

Here, in this work, phthalaldehyde (PAD) was selected as the modifier to react with UiO-66-NH_2_ to fabricate a novel fluorescent sensor, named UiO-66-PSM (Scheme 1). To detect BBH sensitively and selectively, the prepared UiO-66-PSM sensor was designed considering the following two reasons. On the one hand, UiO-66-NH_2_ has unsaturated metal sites (Zr) that can interact with the electrons of the oxygen atoms on BBH. On the other hand, the carboxy of UiO-66-PSM makes it tend to form electrostatic interaction with BBH. The developed UiO-66-PSM material has the advantages of fast response, strong specificity, and high sensitivity as a BBH fluorescence sensor. The effectiveness of the method was verified by the detection of BBH in the traditional Chinese herb *Coptis*. To the best of our knowledge, this is believed to be the first example of a MOF-PSM sensor being used for the analysis of traditional Chinese herbal ingredients.

## 2. Experimental

### 2.1 Materials and reagents

Zirconium chloride (ZrCl_4_), 2-amino-1,4-benzenedicarboxylic acid (NH_2_–H_2_BDC), and PAD were purchased from McLean Reagent (Shanghai, China). Berberine hydrochloride was obtained from Shanghai Yuanye Bio-Technology Co., Ltd. All chemicals were analytical grade or better, and the water used in all experiments was ultra-pure water.

### 2.2 Instrumentation

UV-Vis absorption spectrum was determined by a Shimadzu UV-2550 spectrophotometer. The fluorescence emission spectra were obtained using a Hitachi F-2700 spectrofluorophotometer. Fourier transform infrared (FT-IR) spectra were measured by Shimadzu Tracer-100 FT-IR Spectrometer. Morphological evaluation was carried out by scanning electron microscopy (ZEISS GeminiSEM 300). Energy dispersive X-ray spectroscopy (EDX) was carried out by Rigaku Smartlab 3 KW. Thermogravimetric analysis (TGA) was carried out by TA Q500. Fluorescence decay curves were obtained by Edinburgh FLS1000. Elemental compositions were detected by Thermo Scientific K-Alpha. BET surface area and the pore volume were obtained by Mike ASAP2460.

### 2.3 Synthesis of UiO-66-PSM

UiO-66-NH_2_ was prepared according to the method in the ref. 34. The prepared UiO-66-NH_2_ was dissolved in ethanol (65 ml), PAD (0.6075 g) was added, after which acetic acid was dropped into the UiO-66-NH_2_ solution and sonicated for 5 min. Then the suspension was refluxed at 80 °C for 24 h under a nitrogen atmosphere. The obtained khaki solid was washed with ethanol and dried in a vacuum at 60 °C overnight.

### 2.4 Fluorescence detection of BBH

UiO-66-PSM (2 mg) was dispersed in phosphate buffer solution (PBS, pH = 7.4) and sonicated for 5 min to obtain a homogeneous suspension, after which various concentrations of BBH were added for fluorescence measurement. The fluorescence excitation wavelength was 329 nm and the emission spectra were scanned in the range 349–670 nm.

### 2.5 Real samples preparation

A sample of *Coptis* powder (0.2 g) was passed through a 24 mesh sieve and placed in a conical flask, a mixture of methanol and hydrochloric acid (*V*/*V* = 100 : 1, 50 ml) was added, and the total weight was weighed. Ultrasonic extraction was performed on this mixture for 30 min at room temperature. The mixed solution was weighed again and methanol was added to make up for the lost weight. Then the obtained solution was thoroughly shaken and filtered. The subsequent filtrates (2 ml) were collected and fixed in a 10 ml volumetric flask with methanol. Finally, the yellow extract was shaken well and filtered, the subsequent filtrates were collected once again.

## 3. Results and discussion

### 3.1 Characterization of UiO-66-PSM

**Fig. 1:**
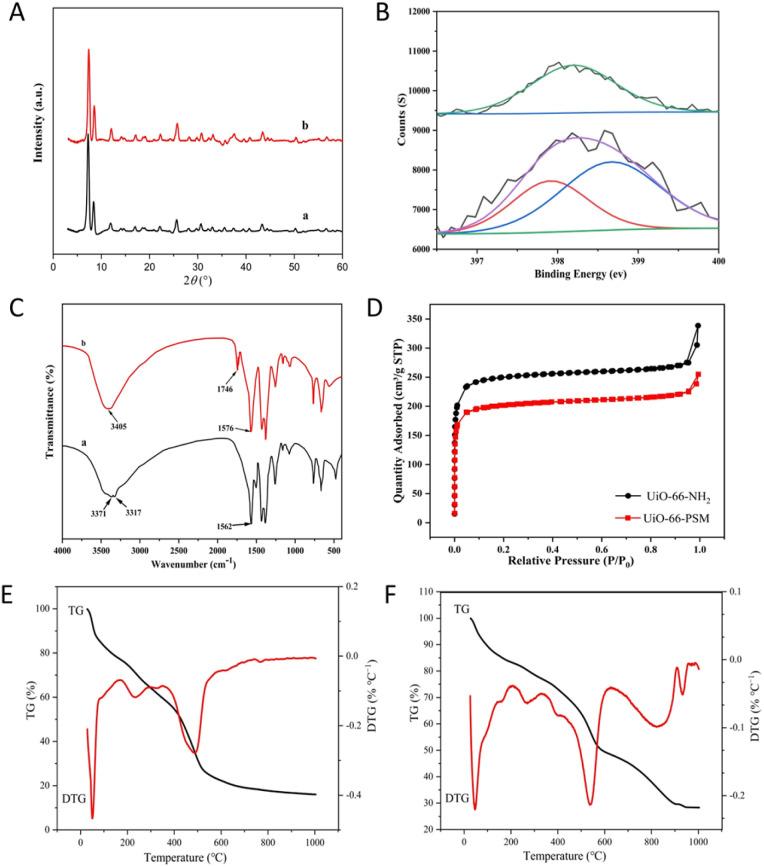
(A) Powder XRD images of (a) UiO-66-NH_2_ and (b) UiO-66-PSM. (B) N 1s spectra of UiO-NH_2_ and UiO-66-PSM. (C) FT-IR spectra of (a) UiO-66-NH_2_ and (b) UiO-66-PSM. (D) N_2_ adsorption–desorption isotherm of UiO-66-NH_2_ and UiO-66-PSM. TG-DTG curves of (E) UiO-66-NH_2_ and (F) UiO-66-PSM.

Scanning electron microscopy (SEM) results displayed the size and morphology of UiO-66-NH_2_ and UiO-66-PSM (Fig. S1†). No significant morphological changes were observed between UiO-66-NH_2_ and UiO-66-PSM. Powder X-ray diffraction (PXRD) was used to confirm the crystalline structure of UiO-66-PSM. As shown in Fig. 1A, the peaks at 7.31, 8.48, and 25.58° corresponded to the crystal planes (111), (200), and (600) respectively.^37^ The results indicated that the crystallinity of UiO-66-NH_2_ was not affected by the introduction of PAD.

The elemental composition of UiO-66-NH_2_ and UiO-66-PSM were studied by X-ray photoelectron spectroscopy (XPS). The signals at 531.08, 399.08, 284.08, and 182.08 eV in the spectra corresponded to O 1s, N 1s, C 1s, and Zr 3d, respectively.^38^ Compared with UiO-66-NH_2_, the additional peak at 399.7 eV in the spectrum of UiO-66-PSM was assigned to amide N (Fig. 1B), suggesting that PAD was successfully assembled on the skeleton of UiO-66-NH_2_. FT-IR spectra of UiO-66-NH_2_ and UiO-66-PSM were presented in Fig. 1C. As can be seen from Fig. 1C(a), the bands at 3317 cm^−1^ and 3371 cm^−1^ belonged to the symmetrical and asymmetrical stretching vibrations of N–H, respectively. However, a broadband replaced the peak of amine functional groups in the UiO-66-PSM spectrum, and a peak at 3405 cm^−1^ was attributed to hydroxyl groups. Furthermore, the increased band at 1746 cm^−1^ belonged to the C=O of the carboxyl group (Fig. 1C(b)).

Emmett–Teller (BET) surface areas and porosities of UiO-66-NH_2_ and UiO-66-PSM were obtained through the measurement of nitrogen (N_2_) adsorption–desorption isotherms (Fig. 1D). The BET surface area of UiO-66-PSM (774 m^2^ g^−1^) was smaller than that of UiO-66-NH_2_ (974 m^2^ g^−1^), which may be due to the introduction of PAD.^39^ According to the IUPAC classification,^40^ the curve was consistent with type I isotherms, N_2_ adsorption on UiO-66-NH_2_ and UiO-66-PSM was dramatically upward at the low pressure region, indicating that they were microporous materials.

Thermogravimetric analysis (TGA) was carried out to study the thermal stability of UiO-66-NH_2_ and UiO-66-PSM in the range of 25–1000 °C. As shown in Fig. 1E, there was an initial weight loss before 340 °C, possibly due to the decomposition of residual solvents in the framework.^41^ The weight loss from 340 to 630 °C was attribute to the thermal dissociation of the UiO-66-NH_2_. As shown in Fig. 1F, the weight loss from 340 to 620 °C may be due to the decomposition of PAD modified on UiO-66-NH_2_. The weight loss from 630 to 960 °C might come from the framework collapse of UiO-66-PSM. The above results indicated that UiO-66-PSM had high thermal stability.

### 3.2 Fluorescence properties of UiO-66-PSM

**Fig. 2:**
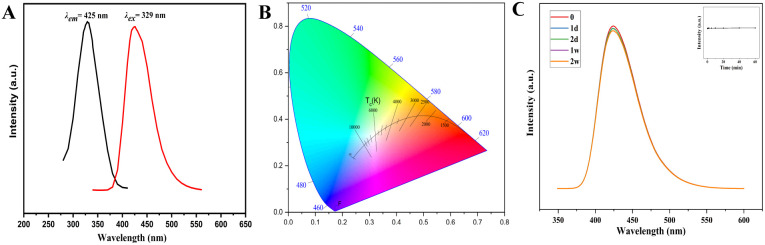
(A) Excitation (black) and emission (red) spectra of UiO-66-PSM. (B) CIE chromaticity diagram of UiO-66-PSM. (C) Inter-day stability of UiO-66-PSM. Inset: changes of the fluorescence intensity of UiO-66-PSM during continuous excitation with a UV lamp for 60 min.

To study the fluorescence properties of the sensor, the excitation and emission spectra of UiO-66-PSM were detected (Fig. 2A). UiO-66-NH_2_ demonstrated a fluorescence emission at 419 nm under excitation at 373 nm, which was ascribed to ligand-to-metal charge transfer (LMCT).^35^ After modification, UiO-66-PSM demonstrated a fluorescence emission at 425 nm under excitation at 329 nm, and exhibited a bright blue fluorescence than UiO-66-NH_2_ under 365 nm UV lamp (Fig. 2B), which may be related to the increase of the conjugation degree and LMCT efficiency of UiO-66-PSM caused by the introduction of PAD (Fig. S2†).

The influence of time and pH on the fluorescence intensity of the UiO-66-PSM sensor was investigated. As shown in Fig. S3,† the fluorescence intensity of UiO-66-PSM firstly increased and then decreased with the increase of pH, and reached the maximum when pH value was 7.4. The internal charge transfer process in the UiO-66-PSM framework was responsible for this pH-dependent fluorescence property. The sensor showed high fluorescence stability within 2 weeks, the change of fluorescence intensity was negligible (Fig. 2C). Moreover, the inset in Fig. 2C showed that the fluorescence of the sensor remained stable after 60 min of continuous exposure to a 365 nm UV lamp.

### 3.3 Sensing of BBH

**Fig. 3:**
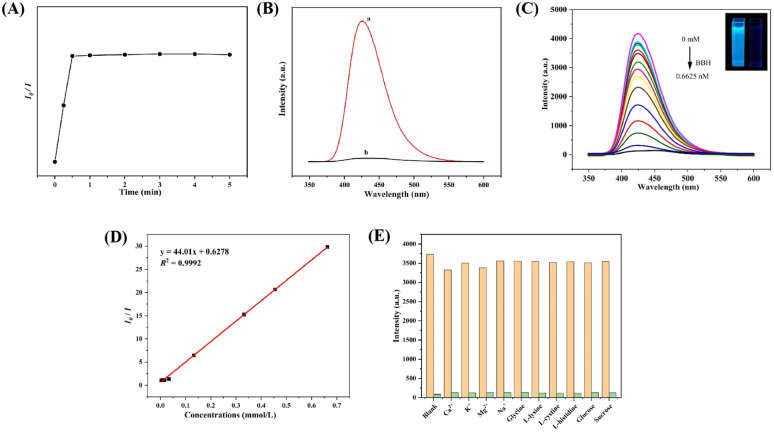
(A) Fluorescence intensity of UiO-66-PSM after adding BBH at different response times. (B) Emission spectra of (a) UiO-66-PSM, (b) UiO-66-PSM + BBH. (C) Fluorescence spectra and (D) fluorescence intensity ratios (*I*_0_/*I*) of UiO-66-PSM after adding various concentrations of BBH (0–0.6625 nM). Inset (C): the photo images of UiO-66-PSM and UiO-66-PSM + BBH under 365 nm UV light. (E) Fluorescence intensity of UiO-66-PSM in different foreign substances in the absence (orange bar) and presence (green bar) of BBH.

Based on the above fluorescence characteristics of UiO-66-PSM, we explored the fluorescence sensing of BBH. The fluorescence response rate of UiO-66-PSM to BBH was studied. As shown in Fig. 3A, the fluorescence intensity of the sensor decreased rapidly after the addition of BBH and stabilized after 30 s. Compared with traditional chromatography, the sensor developed in this work responds faster to BBH, and has great potential in the detection of traditional Chinese herb.

As shown in Fig. 3B, the blue fluorescence of UiO-66-PSM was obviously quenched after the addition of BBH. It can be seen from Fig. 3C that the fluorescence intensity of UiO-66-PSM gradually decreased with the increase of BBH concentration. When 0.1 mM BBH was added, more than 90% of the fluorescence was quenched. The fluorescence intensity ratio (*I*_0_/*I*) of the sensor was linear within the BBH concentration from 3.3 × 10^−3^ to 0.66 mM. The linear regression equation is *y* = 44.01*x* + 0.6278 (*R*^2^ = 0.9992) (Fig. 3D). The limit of detection (LOD) can be calculated using the Stern–Volmer (SV) equation:

1 *I*_0_/*I* = 1 + *K*_sv_ [*Q*]

where *K*_sv_ (M^−1^) is the Stern–Volmer quenching constant, [*Q*] is the concentration of BBH, *I*_0_ and *I* are the luminescence intensities in the absence and presence of BBH, respectively.

According to the above equation, the *K*_sv_ was calculated to be 4.35 × 10^4^ M^−1^, and the LOD was determined to be 0.096 mM based on the 3*σ* rule.^42^ Besides, a comparison between the fluorescence sensor developed in this work and other methods reported for the detection of BBH is listed in Table S1,† indicating that this method has a greater advantage in terms of response speed and linear range.

To better evaluate the selectivity of UiO-66-PSM to BBH, we determined the fluorescence intensity of UiO-66-PSM after adding various potential interferences, including glycine, l-lysine, l-cystine, l-histidine, glucose, sucrose, K^+^, Na^+^, Ca^2+^, Mg^2+^.^6^ The fluorescence quenching was performed by 6.6 × 10^−4^ M BBH and its mixtures with 6.0 × 10^−3^ M other various potential interferences. The above interferences did not cause significant changes in fluorescence intensity (Fig. 3E). Such results indicate that the UiO-66-PSM sensor exhibits merits of specificity and anti-interference ability to BBH, so that the sensor could achieve quantitative detection of BBH.

### 3.4 Analysis of real samples

To investigate the practical application of the developed UiO-66-PSM sensor in the real sample, we determined BBH in the traditional Chinese herb *Coptis*. The determination results of BBH were confirmed by HPLC method (Fig. S4†). As shown in Table 1, the results obtained by this method were close to those obtained by HPLC. *Coptis* spiked with different concentrations of BBH were investigated, the recoveries ranged from 99% to 103.1%, and the RSD was less than 2.0 (*n* = 3). The above results confirm that the UiO-66-PSM sensor is effective and practical for the detection of BBH in traditional Chinese herb.

**Table 1:** Determination results of BBH in *Coptis* samples using the proposed method and confirmed by HPLC method and precision of the proposed method (*n* = 3)^a^

Samples	HPLC (mM)	Spiked (mM)	Found (mM)	Accuracy		Precision
				Recovery (%)	RSD (%)	RSD (%)
*Coptis*	0.104	—	0.117	—	—	1.6
		10	10.2	101.1	1.5	
		20	19.9	99.0	2.0	
		30	31.0	103.1	2.5	

a HPLC: the amount of BBH determined by HPLC.

### 3.5 Investigation of fluorescence sensing mechanism

**Fig. 4:**
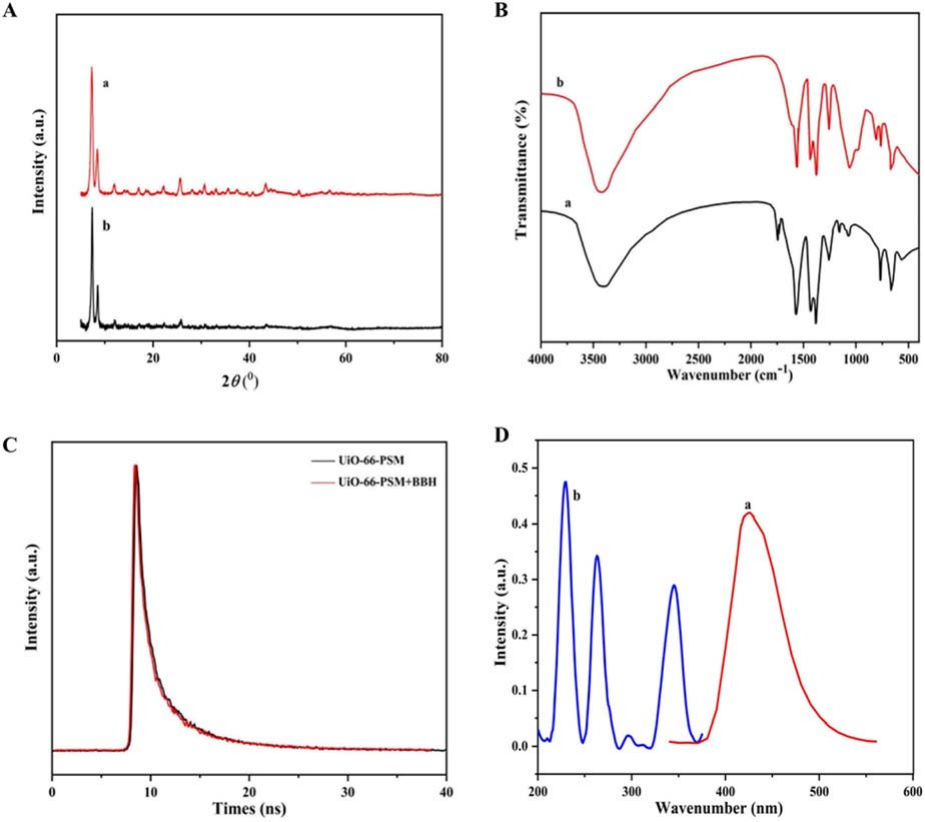
(A) PXRD patterns, (B) FT-IR spectra of the UiO-66-PSM (a) before and (b) after adding BBH. (C) Fluorescence lifetime of UiO-66-PSM in the (black) presence and (red) absence of BBH. (D) (a) Emission spectrum of UiO-66-PSM and (b) UV-vis absorption spectrum of BBH.

In general, there are several main reasons for fluorescence quenching in MOF-based sensors: collapse of the MOF framework,^43^ fluorescence resonance energy transfer (FRET),^44^ and photo-induced electron transfer (PET).^45^ As can be seen from Fig. 4A, the diffraction peaks in the PXRD spectrum of UiO-66-PSM were approximately unchanged after the introduction of BBH, implying that the structure of UiO-66-PSM was not destroyed during the quenching process. FT-IR spectra of the sensor before and after the addition of BBH were shown in Fig. 4B. After adding BBH, the peak of C=O had a blue shift from 1657 cm^−1^ (curve a) to 1620 cm^−1^ (curve b), implying the interaction between the carboxylic acid group of UiO-66-PSM and the N of BBH. Therefore, the interactions could be speculated as follows: the coordination between the oxygen atoms of BBH and unsaturated metal sites (Zr); the acid–base interaction between the carboxylic acid group on UiO-66-PSM and the N of BBH. Due to BBH was immobilised and the distance between the ligand and BBH was close, the energy absorbed by the ligand could be transferred to BBH, which reduced the ligand-to-metal energy transfer efficiency and led to the quenching effect on the fluorescence intensity of UiO-66-PSM.^46^ Thus, the fluorescence quenching mechanism of UiO-66-PSM should be PET.

We further investigated the fluorescence quenching mechanism of UiO-66-PSM towards BBH. The fluorescence emission spectrum of UiO-66-PSM and the UV-vis absorption spectrum of BBH were measured. As shown in Fig. 4D, the UV-vis absorption of BBH did not overlap with the emission of UiO-66-PSM, thus the FRET mechanism was excluded.

The mechanism of fluorescence quenching can be divided into dynamic quenching and static quenching.^47^ Time-resolved fluorescence decay experiments were performed. As shown in Fig. 4C, the fluorescence lifetimes of UiO-66-PSM in the presence and absence of BBH were 8.69 ns and 8.58 ns, respectively. The fluorescence lifetime of the sensor was essentially unaffected by the introduction of BBH, demonstrating that the detection mechanism was static fluorescence quenching.

## 4. Conclusion

In summary, we designed and synthesized a fluorescent sensor (UiO-66-PSM) in a relatively short time, and applied it to the quantitative detection of BBH. The developed sensor exhibited high sensitivity (0.096 mM), fast response time (30 s) and excellent selectivity. In addition, the sensor was successfully applied to detect BBH in the traditional Chinese herb *Coptis*. It is expected that the MOF-based sensor material will have great application prospects in the detection of other ingredients in traditional Chinese herb.

## Author contributions

Wei Liu: conceptualization, data curation, methodology, formal analysis, investigation, visualization, writing – original draft. Shuang Wu: methodology, formal analysis. Tian-Xia Sun: validation. Jing Bai and Ying Yang: resources. Wen-Hui Lian: writing – review & editing. Yu Zhao: funding acquisition, project administration.

## Conflicts of interest

There are no conflicts to declare.

## Supplementary Material

RA-014-D3RA07054A-s001
