# Supplementary material for: Post-synthetic modified luminescent metal–organic framework for the detection of berberine hydrochloride in a traditional Chinese herb
Source: RSC Adv. 2024 Jan 2;14(1):602–7. doi: 10.1039/d3ra07054a (PMC10759037; doi:10.1039/d3ra07054a)
Supplement: RA-014-D3RA07054A-s001 [file RA-014-D3RA07054A-s001.pdf]

Electronic Supplementary Material (ESI) for RSC Advances.
This journal is © The Royal Society of Chemistry 2023

Supplementary Material

Post-synthetic modified luminescent metal-organic framework for the detection of berberine hydrochloride in traditional Chinese herb

Wei Liu,[a] Shuang Wu,[b] Tian-Xia Sun,[b] Jing Bai,[c] Ying Yang,[c] Wen-Hui Lian*[b] and Yu Zhao*[b]

[a] *College of Pharmacy, Changchun University of Chinese Medicine, Changchun 130017, P. R.China*

[b] *Jilin Ginseng Academy, Changchun University of Chinese Medicine, Changchun 130017, P. R.China*

[c] *Jilin Ji Test Technology Co. LTD, Changchun 130017, P. R.China*

Corresponding author: Wen-Hui Lian; Yu Zhao

Email address: lianwenhui1014@126.com; cnzhaoyu1972@126.com

CONTENTS

Figs. S1-S4··**SI-2**

Table S1···**SI-4**

References··**SI-4**

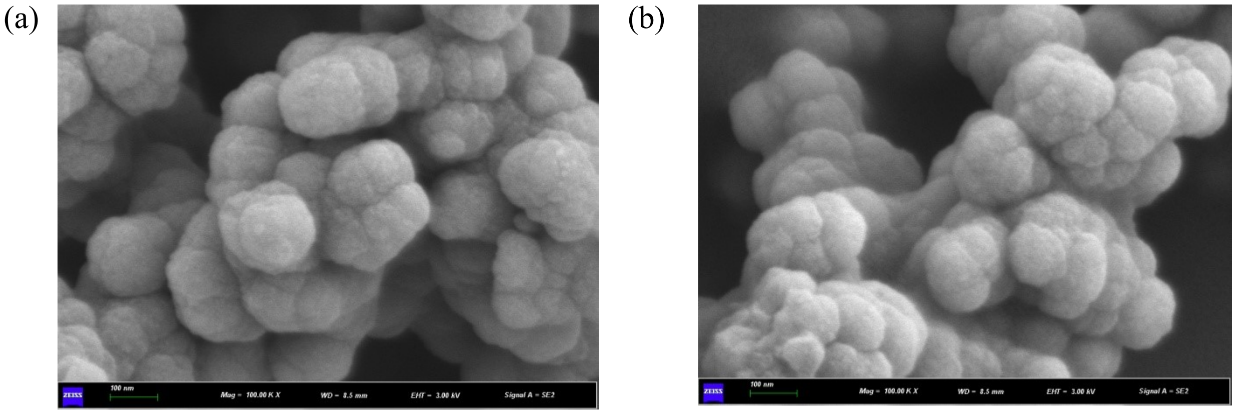

Fig. S1 SEM images of (a) UiO-66-NH_2 and (b) UiO-66-PSM.

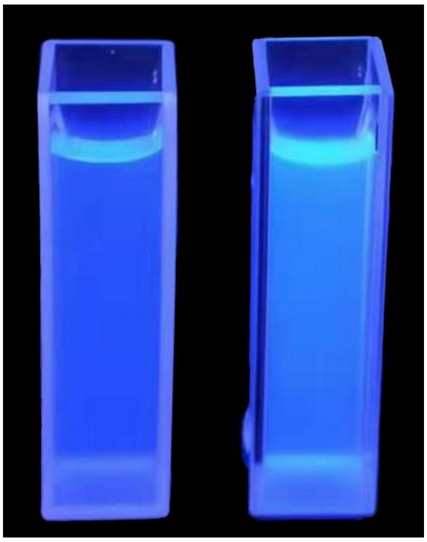

Fig. S2 Photo image of UiO-66-NH_2 and UiO-66-PSM under 365 nm UV light.

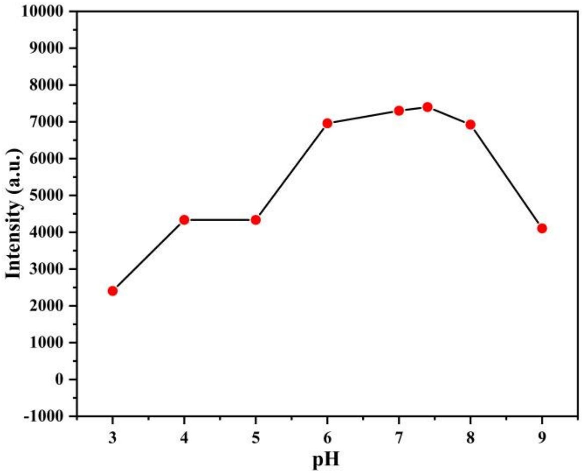

Fig. S3 Fluorescence intensity of UiO-66-PSM at different pH values.

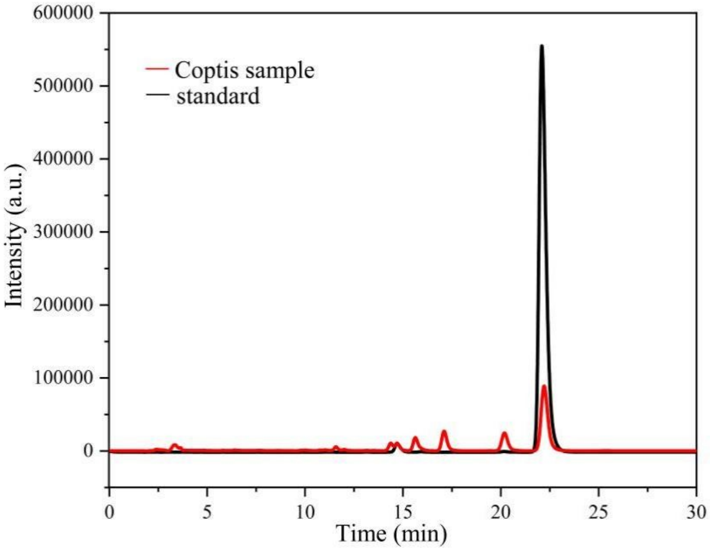

Fig. S4 HPLC−UV chromatograms of BBH standard solution and *Coptis* sample.

Table S1 Comparison of the present method with other reported methods for BBH determination

Methods	Linear range	LOD	Response time	Ref.
HPLC	10–2360 ng/mL	0.6 ng/mL	2.5 min	1
Colorimetry	0.05–0.4 μmol/L	13 nμmol/L	5 min	2
Electrochemistry	0.25–30 μmol/L	140 nμmol/L	30s	3
Fluorescence	1–100 μmol/L	75 nμmol/L	2 min	4
Fluorescence	0.5–30 μmol/L	50 nμmol/L	10 min	5
Fluorescence	0–200 μmol/L	57.35 nμmol/L	—	6
Fluorescence	0.5–320 μmol/L	78 nμmol/L	15 min	7
Fluorescence	1×10^{3}–1×10^{6} μmol/L	0.3 nμmol/L	—	8
Fluorescence	10 –130 μmol/L	3.5 nμmol/L	10 min	9
Fluorescence	3.3×10^{-6}–6.6×10^{-4}μmol/L	96 nμmol/L	30 s	This work

References

1. J. Feng, W. Xu, X. Tao, H. Wei, F. Cal, B. Jiang and W. Chen, *J. Pharm. Biomed. Anal.*, 2010, **53**, 591-598.
2. J. Ling, Y. Sang and C. Z. Huang, *J. Pharm. Biomed. Anal.*, 2008, **47**, 860-864.
3. A. Geto, M. Pita, A. L. De Lacey, M. Tessema and S. Admassie, *Sens. Actuators B-chem*, 2013, **183**, 96-101.
4. A. Wen, X. Peng, P. Zhang, Y. Long, H. Gong, Q. Xie, M. Yue and S. Chen, *Anal. Bioanal. Chem.*, 2018, **410**, 6489-6495.
5. J.-M. Liang, F. Zhang, Y.-L. Zhu, X.-Y. Deng, X.-P. Chen, Q.-J. Zhou and K.-J. Tan, *Spectroc Acta Pt A-Molec. Biomolec. Spectr.*, 2022, **275**, 121139.
6. M. Liu, X. Du, K. Xu, B. Yan, Z. Fan, Z. Gao and X. Ren, *J. Anal. Sci. Technol.*, 2021, **12**, 11-20.
7. J. Xiong, L. Yang, L. X. Gao, P. P. Zhu, Q. Chen and K. J. Tan, *Anal. Bioanal. Chem.*, 2019, **411**, 5963-5973.
8. S. Liang, Y. Kuang, F. Ma, S. Chen and Y. Long, *Biosens. Bioelectron.*, 2016, **85**, 758-763.
9. F. Yan, Z. Sun, S. Luo and H. Zhang, *Dyes Pigment.*, 2021, **192**, 109446-109454.
